# Spatiotemporal Patterns of the Use of Urban Green Spaces and External Factors Contributing to Their Use in Central Beijing

**DOI:** 10.3390/ijerph14030237

**Published:** 2017-02-27

**Authors:** Fangzheng Li, Fen Zhang, Xiong Li, Peng Wang, Junhui Liang, Yuting Mei, Wenwen Cheng, Yun Qian

**Affiliations:** 1 School of Landscape Architecture, Beijing Forestry University, Beijing 100083, China; fangzhengli@bjfu.edu.cn (F.L.); fenzhang@bjfu.edu.cn (F.Z.); yutingmei@bjfu.edu.cn (Y.M.); Qianyun@bjfu.edu.cn (Y.Q.); 2 Beijing Laboratory of Urban and Rural Ecological Environment, Beijing 100083, China; 3 Beijing Tsinghua Tongheng Urban Planning & Design Institute, Beijing 100085, China; wangpeng@thupdi.com (P.W.); liangjunhui@thupdi.com (J.L.); 4 Department of Landscape Architecture, Texas A&M University, College Station, TX 77840, USA; cheng1989@tamu.edu

**Keywords:** urban green spaces, spatiotemporal patterns, visitor distribution, external contributing factors, geographical detectors

## Abstract

Urban green spaces encourage outdoor activity and social communication that contribute to the health of local residents. Examining the relationship between the use of urban green spaces and factors influencing their utilization can provide essential references for green space site selection in urban planning. In contrast to previous studies that focused on internal factors, this study highlights the external factors (traffic convenience, population density and commercial facilities) contributing to the use of urban green spaces. We conducted a spatiotemporal analysis of the distribution of visitors in 208 selected green spaces in central Beijing. We examined the relationship between the spatial pattern of visitor distribution within urban green spaces and external factors, using the Gini coefficient, kernel density estimation, and geographical detectors. The results of the study were as follows. The spatial distribution of visitors within central Beijing’s green spaces was concentrated, forming different agglomerations. The three examined external factors are all associated with the use of green spaces. Among them, commercial facilities are the important external factor associated with the use of green spaces. For the selection of sites for urban green spaces, we recommend consideration of external factors in order to balance urban green space utilization.

## 1. Introduction

Urban green spaces have significant social, ecological, and cultural value. Several studies have demonstrated that urban green spaces offer benefits relating to individuals’ health and wellbeing such as relaxation [1], recreational opportunities [2,3], and a connection to nature [1]. Other studies have also confirmed that urban green spaces are key components of ecosystem services that evidently contribute to the mitigation of high temperatures [4,5,6,7], abatement of air pollution [8], noise reduction [5], the provision of wildlife habitats [9,10,11,12], and the prevention of floods and soil erosion [13]. Furthermore, studies have revealed important benefits associated with cultural services provided by urban green spaces such as landscape aesthetics, outdoor recreation, and spiritual and cultural values [14,15].

Although large areas and expanses of green space exist in megacities such as Beijing, Tokyo, and New York, not all urban green spaces can be effectively used by the public. The planning of urban green spaces requires a thorough understanding of the factors that promote their use. Urban green spaces are places where the public can engage in physical activities. Therefore, they have beneficial impacts on human health [16]. Measures to enhance the use of urban green spaces can encourage people to be more physically active. Consequently, identifying the reasons why people visit green spaces can facilitate the planning of urban green spaces aimed at fostering an active lifestyle that promotes good health. A considerable number of studies have examined the factors associated with people’s utilization of urban green spaces [1,16]. These spaces were found to be important for relaxation, mental restoration, and physical activities, or for simply socializing outdoors [17]. The type of use of green spaces has been associated with their properties and configurations [18]. For example, studies have found that physical activities are associated with the properties of green spaces such as their tree, water, and light components [19,20,21]. Recent studies have also shown that the presence of a particular type and feature in green space have mixed effects on the use of urban green spaces. Other studies have shown that landmarks with solid edges within urban green spaces (e.g., a steep slope or trees) may be associated with numbers of people sitting on the grass [22]. Additionally, vegetation quality has been found to be correlated with people’s utilization of urban green spaces [23]. Studies that have focused on internal factors influencing the use of urban green spaces have yielded similar findings. 

However, a critical research direction relates to external factors contributing to people’s use of urban green spaces. External factors refer to influencing factors located around green spaces [24] without consideration of the factors related to urban green spaces themselves (e.g., plants, water and facilities). Some studies have examined the relationship between accessibility of urban green spaces and their utilization ratio [25]. Many works have shown that factors related to accessibility included distance [26,27], time spent, and types of available transportation [27]. A recent study revealed that the attractiveness of facilities in the vicinity of urban green spaces also has a bearing on the number of visitors visiting these spaces [28]. Moreover, surveys have shown that demographic factors were determinants of the social context [29,30,31]. Users’ socioeconomic backgrounds [18,31,32] also had a strong correlation with their use of urban green spaces. These studies have laid the foundation for understanding the correlation of external factors with the relationship between urban green spaces and the spatial distribution patterns of visitors in these spaces. However, a question that remains to be addressed concerns the degree to which the external factors influence the spatial distribution patterns of visitors in urban green spaces. Because of limited resources, most surveys have only been conducted at a few selected sites [18,20,23,32,33]. Moreover, the paucity of studies that have considered all external contributing factors has made the direct comparison of these factors a difficult task. Evidently, there is a need to apply a variety of research methods for obtaining data relating to more urban green spaces compared to previous studies to provide a satisfactory response to this question, thus improving the planning and site selection of urban green spaces.

For this study, we selected 208 urban green spaces in central Beijing as our study sites, attempting to gain an understanding of external factors that were associated with visitors’ spatial distribution patterns within these spaces. Previous studies have been concerned with the effect of each external factor. However, they failed to compare the external factor with quantitative analysis. A lingering question is: Which are the most important external factors that influence people to visit urban green spaces? Based on previous studies, we divided these external factors located around the green spaces into three categories: transportation convenience [25,28,34], population density [30,31], and commercial facilities [35]. The study’s main objectives were: (1) to study the features of visitors’ spatiotemporal patterns in urban green spaces; (2) to determine the relative importance of external factors that associated with visitors’ spatiotemporal distribution in urban green spaces; and (3) to recommend measures for balancing urban green spaces utilization in megacities such as Beijing, Tokyo, or New York.

## 2. Materials and Methods

### 2.1. Study Area

As China’s capital, Beijing is a typical megacity. Its boundaries lie between the longitudes of 115.25° E and 117.30° E and the latitudes of 39.28° N and 41.25° N. According to the Beijing Statistical Information Net, at the end of 2015, the area under the city’s administration was 16,410 km^2^ and its built-up area was 1386 km^2^. The total population of Beijing at the end of 2015 was 21.705 million. For this study, we selected Beijing’s central districts (Dongcheng, Xicheng, Haidian, Chaoyang, Fengtai, and Shijingshan) as our study areas. 

### 2.2. Data Sources

#### 2.2.1. Classification of Urban Green Space

The objects of the study, namely urban green spaces and country parks, were categorized prior to conducting the study. “China’s Urban Green Space Classification Standard” [9] defines urban green spaces as “green area with certain recreational facilities and service facilities which are open to the public; they are mainly used for recreation with functions of ecology, environment improvement, disaster prevention and reduction.” The five main categories of urban green spaces are: comprehensive parks, community parks, theme parks (e.g., children’s parks, zoos, botanical gardens, historical gardens, and famous scenic parks), belt-shaped parks, and roadside green spaces. Comprehensive parks, with rich recreational facilities, are large in scale and are suitable for outdoor activities. Community parks mainly offer certain physical activities as services for residents. Theme parks entail specific theme-based activities and facilities. Belt-shaped parks are always narrowly spread alongside urban roads, city walls, and waterfronts. Roadside green spaces are small in size and are mainly used as whistle-stops. Because the influence of roadside green space on the density of the spatial distribution of visitors is minimal, we excluded this category of urban green space from our study. 

The second category of urban green space included in the study was the country park. In most urban areas of China, country parks have been developed to sustain ecosystems and the environment. Beijing’s country parks are unique as they play a similar role to that of other urban green spaces. For example, they provide a wide range of recreational activities and consequently have considerable correlation with the density of the spatial distribution of visitors.

These five types of urban green spaces: comprehensive parks, community parks, theme parks, belt-shaped parks, and country parks) were selected as our objects of study.

#### 2.2.2. Data Sources

We collected three sets of data. The first comprised spatial distribution data relating to 208 urban green spaces. These data were collected to analyze the distribution characteristics of urban green spaces in central Beijing. The second dataset comprised information on the number of visitors in urban green spaces to assess the spatiotemporal pattern of distribution of visitors. The third dataset, which focused on transportation convenience, population density, and commercial facilities, was collected to explore the relationship between the pattern of spatial distribution of visitors in urban green spaces and external factors contributing to their use.

The data on urban green spaces were obtained using point of interest (POI) crawler technology based on a map of Baidu produced in 2015 [36]. The data on the number of visitors in urban green spaces were crawled through a WeChat public number termed Tencent easygo (WeChat ID: easygo-qq) over the period from 19 July 2015 to 2 August 2015 (including three weekdays and two weekends). The number of visitors was reflected in the point data on visitors. The total number of visitors was 6,104,811. Each point represented an area of 27 m × 27 m. The data relating to metro and bus stations and streets were used to assess transportation convenience. These were also obtained from the Baidu map of 2015 using the POI crawler technology. Street populations, derived from census data, were obtained by tabulating Beijing’s urban population in 2015. Street populations also encompassed populations residing within a range of 1 km from urban green spaces. The data on commercial facilities were also obtained through the POI crawler technology based on the Baidu map of 2015.

ArcGIS 10.0 software (Environmental Systems Research Institute, Inc., Redlands, CA, USA) was applied to vector handle central Beijing with a map of Beijing produced in 2015 and this was considered as a working base map (Beijing Geodetic Coordinate System 1954). Base map hierarchical information included the main traffic lines (line layer) and administrative divisions (polygon layer, including district and sub-district layers), with the most recent metro lines and stations (point layer) obtained from the Baidu map.

### 2.3. Methods

In this study, the number of visitors in urban green spaces at the end of 2015 was collected as statistical data. Based on these data, the spatial aggregation of visitors was calculated by applying the Gini coefficient and kernel density estimation (KDE) methods. Further research was conducted to analyze the relationship between the spatial distribution of visitors in urban green spaces and external factors contributing to their use by applying geographical detectors.

#### 2.3.1. Gini Coefficient

The Gini coefficient method was used to measure the spatial aggregation of visitors and the distribution of urban green spaces. The Gini coefficient, which can be used as a measure of inequality, has been widely applied across various contexts such as energy, credit availability, income, health care, and wealth [37]. Because the Gini coefficient can be used to measure inequality among values of frequency distribution based on discrete and continuous distributions [37], it can describe the aggregation level of clusters. The Gini coefficient (*G*) was expressed by the following equation:

(1) $$G=\frac{1}{2n^{2}\bar{s}}\sum_{i=1}^{n}\sum_{j=1}^{n}\left|s_{i}-s_{j}\right|$$

where *G* is the Gini coefficient, n is the number of streets, $\bar{s}$ is the average point density of urban green spaces in all streets, and $s_{i}$ and $s_{j}$ are the point densities of urban green spaces in sub-districts *i* and *j* (the number of urban green spaces/the area of streets in km^2^). A larger Gini coefficient corresponds to greater inequality. Absolute equality is denoted by a Gini coefficient below 0.2, relative equality corresponds to a Gini coefficient within a range of 0.2–0.3, proper inequality is indicated by a Gini coefficient ranging between 0.3–0.4, a high degree of inequality is denoted by a Gini coefficient between 0.4 and 0.5, and a Gini coefficient above 0.5 represents severe inequality [37,38,39].

#### 2.3.2. Kernel Density Estimation

KDE is a effective measurement used to describe the spatial structure of the density of visitors within each of the selected urban green spaces. Kernel density entails a statistical method used for estimating a continuous and smooth distribution from a finite set of observed points [40]. KDE uses a “moving window” that weights events within its sphere of influence according to their distance from each grid to estimate the intensity within each grid [41]. The underlying rationale of KDE is that each data point (X1, X2, . . . , Xn) of a sample of size n from a random variable with a unknown probability density function *fx(x*) is replaced by a specified function *K*(·), which is the kernel, centered at each data point Xi, and with a scaling parameter *h* referring to the bandwidth or smoothing constant. The sum of the kernel functions is scaled to produce a smooth curve, which is a unit area. This is the density estimate of *fx(x*) at point *x*, and is expressed as follows:

(2) $$f_{n}(\chi )=\frac{1}{nh}\sum_{i=1}^{n}k(\frac{\chi -\chi_{i}}{h})$$

where *K*( ) is the kernel function, *h* is the bandwidth (*h* > 0),and n is the number of known points in the bandwidth. 

#### 2.3.3. Geographical Detectors

Geographical detectors constitute a spatial statistical method used to quantitatively investigate the effects of external factors on the use of urban green spaces. Geographical detectors were first introduced by Wang (2010) as a means of identifying and describing the relationship between risk factors and certain diseases [42]. They have subsequently been widely applied to test the effects of potential driving factors on geographical phenomena [43]. A factor detector entailing the determinant of statistical power can be used to assess whether a particular geographical factor is associated with diverse spatial distribution. Consequently, the relative importance of the relevant factors can be quantitatively indicated. $P_{D,H}$ is defined to express the relative importance of the factors [43].

In this study, geographical detectors were used to examine the correlation of external factors with the density of visitors in urban green spaces representing the use of urban green spaces. To accomplish this objective, it was necessary to first superimpose the distribution of the density of visitors in urban green spaces on to the geographical stratum of the external factors. The data of transportation convenience, population density, and commercial facilities located around the green spaces of Beijing in 2015 were classified and mapped. As previously mentioned, transportation convenience was calculated and categorized into three levels according to the numbers of metro stations, bus stations, and main roads. These categories were: levels 1 (0–2), 2 (3–6), and 3 (7–11) for metro stations; levels 1 (0–16), 2 (17–37), and 3 (38–105) for bus stations; and levels 1 (0–25), 2 (26–51), and 3 (52–110) for main roads. Based on their permutations and combinations, these three elements of transportation, considered collectively, were subsequently grouped into classes of transportation convenience. Class 1 transportation convenience entailed more than two elements belonging to level 1. Class 2 transportation convenience entailed either more than two elements belonging to level 2, or one element each belonging to levels 1 and 2, respectively. Class 3 entailed the remaining elements. The ArcGIS software and the natural breakpoint method were used to calculate and divide population density into nine categories [44]. ArcGIS software and the natural breakpoint method were also used to measure distances between urban green spaces and commercial facilities located nearest to them to calculate and classify commercial facilities. Five categories of commercial facilities were identified. The dispersion variance between the distribution of density of visitors in urban green spaces and the sub-regions of the attributes external factors were denoted as $\sigma^{2}$. The power of external factors in relation to the distribution of the density of visitors in urban green spaces was expressed by the following equation:

(3) $$P_{D,H}=1-\frac{1}{n\sigma_{H}^{2}}\sum_{i=1}^{m}n_{D,i}\sigma_{H_{D,i}}^{2}$$

where *D* is the influencing factor (e.g., transportation convenience, population density, or commercial facilities), *H* is the density of visitors in urban green space, $P_{D,H}$ is the effect of *D* on *H*, *n* and $\sigma^{2}$ are the number and variance of the samples, respectively, *m* is the classification number of an index, and *n_D,I_* is the sample number of *D*. Within a range of values from 0 to 1, the larger the $P_{D,H}$ value is, the greater the factor’s effect on the density of visitors within this particular category of urban green space. 

## 3. Results and Discussion

### 3.1. Spatial Distribution Characteristics of Urban Green Spaces

**Figure 1:**
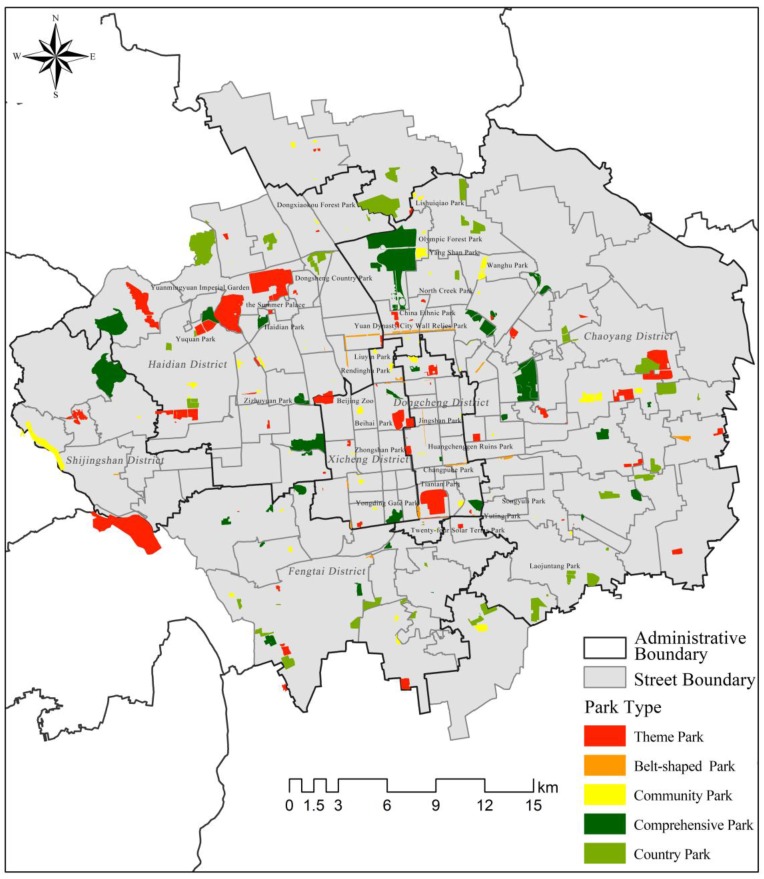
Distribution of urban green spaces in central Beijing in 2015.

POI crawler technology was applied to identify 208 urban green spaces (comprehensive parks, community parks, theme parks, belt-shaped parks, and country parks) in central Beijing (Table 1). POI data usually includes spatial information such as longitudes and latitudes and information such as names and categories. Applying the POI data, we conducted extensive fieldwork in our study area to ensure the accuracy of the locations and boundaries of all of the selected urban green spaces. Spatial analysis was subsequently conducted using the ArcGIS 10.0 software to analyze the spatial distribution characteristics of these spaces (Figure 1).

To identify the spatial distribution characteristics of urban green spaces in central Beijing, we calculated the quantities and areas of the five kinds of urban green spaces (Table 1) and then conducted an analysis entailing the Gini coefficient (Table 2). As shown in Table 1, the Gini coefficient was highest for community parks, with a value of 0.466. Figure 1 presents the overall distribution of community parks. We found that community parks were mainly distributed near large communities such as the Anhuili, Huizhongli, and Yaojiayuan communities. This suggests that geographical aggregation was a key factor in the creation of community parks. The Gini coefficient of theme parks was also relatively high at a value of 0.414. Furthermore, theme parks were mainly distributed along Second Ring Road as well as in Beijing’s western suburbs. Therefore, theme parks could easily form geographical aggregation. 

Comprehensive parks were relatively well-distributed in the central part of the city. However, there were variations between districts, with comprehensive parks being aggregated in Chaoyang District and, to a lesser degree, in Haidian District. On the other hand, belt-shaped parks were few in number and reflected in the lowest Gini coefficient for this category of green space. Country parks typically encircled Five Ring Road. Although there were 40 country parks in central Beijing, they did not connect with each other to form a completely closed “Park Ring”.

### 3.2. Spatiotemporal Pattern of Distribution of Visitors in Urban Green Spaces

#### 3.2.1. Temporal Distribution Characteristics of Visitors

To analyze the temporal distribution characteristics of visitors, we divided a day into eight periods according to the daily life rhythms of residents. Based on these time periods, we analyzed numbers of visitors in the 208 urban green spaces to assess temporal variations as well as differences between weekdays and weekends. 

**Figure 2:**
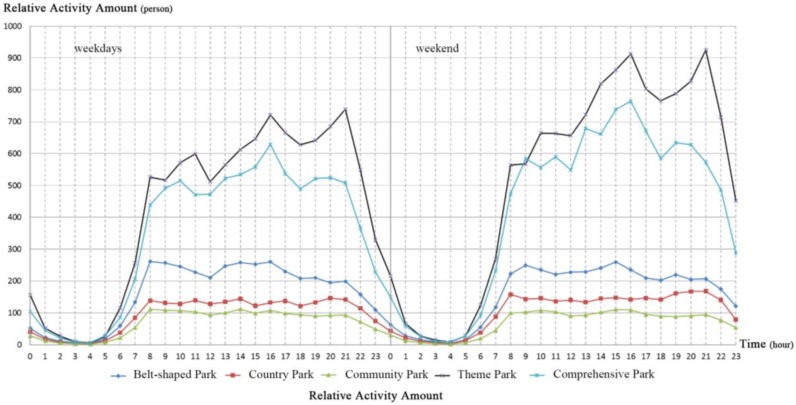
Numbers of visitors in urban green spaces in central Beijing in 2015.

Figure 2 presents evident temporal variations in numbers of visitors during weekdays and weekends. Among the five kinds of urban green spaces, theme parks evidenced the highest number of visitors. During weekdays, there were four peak time periods for visitor numbers in theme parks: 7–8 a.m., 10–11 p.m., 3–4 p.m., and 8–9 p.m. During weekends, peak values of visitor numbers in theme parks occurred during two periods (3–4 p.m. and 8–9 p.m.). Numbers of visitors on weekends exceeded those on weekdays. Temporal variations in numbers of visitors in comprehensive and theme parks were similar. Visitor numbers in comprehensive parks peaked between 3 and 4 p.m. both on weekdays and weekends. Moreover, visitor numbers in both spaces fell during weekdays. Similar variations in visitor numbers were found for belt-shaped parks on weekdays or weekends. This may be attributed to the primary use of belt-shaped parks as corridors that visitors pass through. Visitor numbers in country parks presented the same pattern. Specifically, peak visits on both weekdays and weekends occurred over the period from 8 to 9 p.m. This finding indicates that country parks in Beijing are gradually becoming significant recreation destinations for nearby residents on summer nights (8–10 p.m.). Visitor numbers in community parks were relatively consistent on weekdays and weekends. This suggests that visitors were visiting community parks with high walking accessibility and were not limited by time.

#### 3.2.2. Characteristics of Spatial Agglomerations of Visitors

The spatial agglomeration of visitors was analyzed based on the Gini coefficient calculated for numbers of visitors in urban green spaces. As shown in Table 3, the Gini coefficients of visitor numbers in the five kinds of urban green spaces were higher on weekdays than on weekends. This finding suggests a higher level of spatial agglomeration of visitors on weekdays than on weekends and can be attributed to the wider range of urban green spaces available to visitors who are not limited by time and space on weekends. This translated into lower levels of spatial agglomeration of visitors during weekends. The Gini coefficient for visitor numbers on weekdays was the highest for comprehensive parks. A comparison of this value with the Gini coefficient for the distribution of urban green spaces, shown in Table 2, reveals that comprehensive parks are relatively well-distributed in central Beijing. Furthermore, most of the large office buildings in Beijing are bordered by comprehensive parks. Therefore, visitors from these work spaces find it convenient to use comprehensive parks. Thus, the level of spatial agglomeration of visitors in comprehensive parks located around large working places was relatively high even during weekdays. However, theme parks evidenced the highest level of spatial agglomeration of visitors on weekends. Table 2 shows a high level of geographical aggregation for theme parks. Moreover, these parks are characterized by their specific themes such as the Summer Palace and Haidian Park. This suggests that visitors without limitations of time and space intentionally chose theme parks according to their specific theme and its appeal for recreation purposes.

#### 3.2.3. Spatial Structure of Visitor Distribution

**Figure 3:**
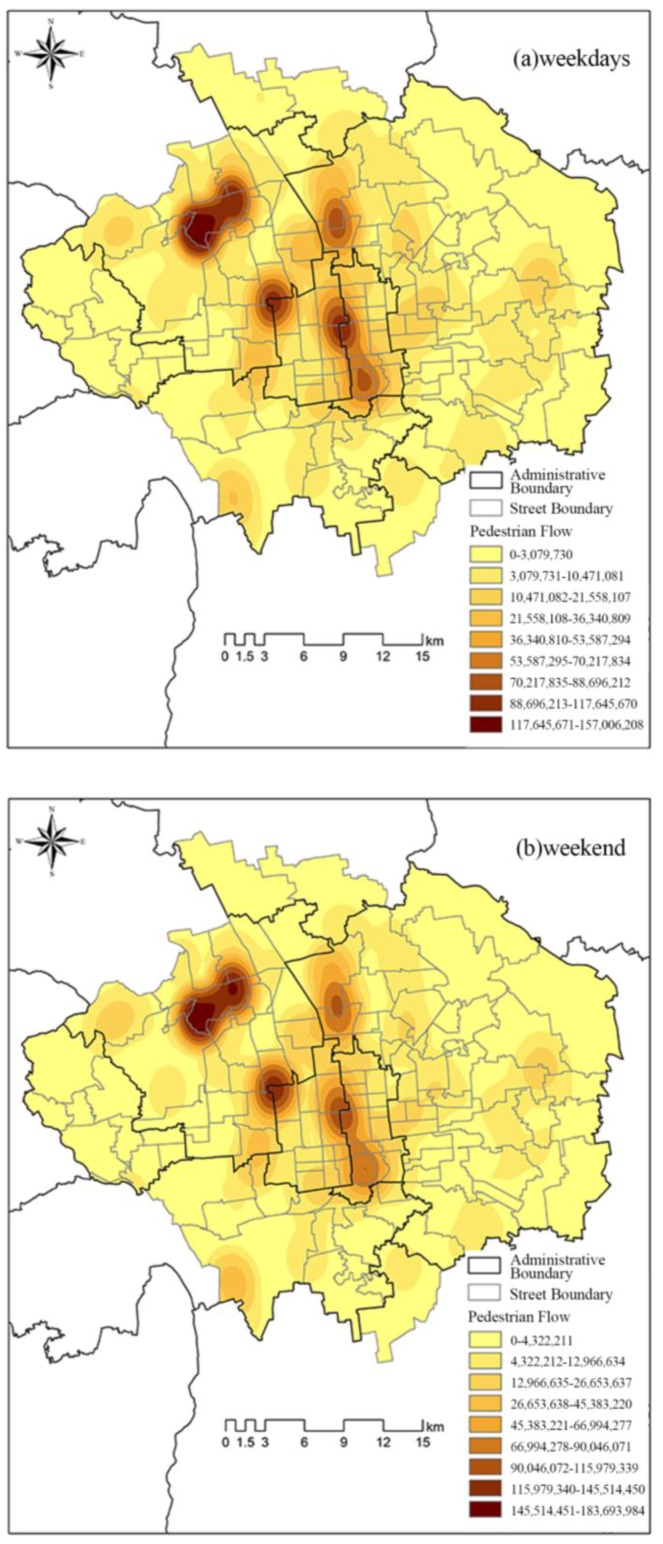
Kernel density relating to visitor numbers on (**a**) weekdays and (**b**) weekends.

The KDE method was applied to analyze the locations of spatial agglomerations of urban green spaces as well as their distribution characteristics. As shown in Figure 3, the spatial pattern of the distribution of visitors and the KDE value for visitor numbers on weekdays and weekends was clearly reflected. Core agglomeration was used to reflect the gathering places where the high-density spots of visitors in green spaces were located. The spatial pattern of the distribution of visitors on weekdays and weekends showed similar variations, with both entailing two dual-core and two single-core agglomerations. Dual-core agglomerations were where two gathering areas of the high-density spots of visitors were close together. However, the overall values for the kernel density for visitor numbers on weekdays and weekends were different. The dual-core agglomeration with the highest kernel density of visitor numbers occurred in Haidian District which encompasses four theme parks (the Summer Palace, Yuanmingyuan Imperial Garden, Yuquan Park, and Wanquan Cultural Park), two comprehensive parks (Haidian Park and Yudong Park) and one country park (Yudong Country Park). The second dual-core agglomeration was found along Second Ring Road, formed by five theme parks (Tiantan Park, Zhongshan Park, Beihai Park, Jingshan Hill Park, and Twenty-four Solar Terms Park), three belt-shaped parks (Yongding Gate Park, Yu Ting Park, and Huangchenggen Ruins Park) and one community park (Changpuhe Park). Theme parks were the key components of the structures of both of these two dual-core agglomerations. Moreover, the values of the kernel density of visitor numbers for these two dual-core aggregations were higher on weekends than on weekdays. Two additional single-core agglomerations were identified in Haidian and Chaoyang Districts, respectively. The single-core agglomeration in Haidian District comprised one theme park (Beijing Zoo) and one comprehensive park (Zizhuyuan Park). It is noteworthy that the density of visitors in this single-core agglomeration remained at almost the same level on weekdays and weekends. The second single-core agglomeration in Chaoyang District mainly comprised the Olympic Forest Park, which is surrounded by some theme parks (e.g., China Ethnic Park), community parks (e.g., Yang Shan Park) and country parks (e.g., Dongxiaokou Forest Park, and Dongsheng Style Park). Moreover, the highest density of visitors for this single-core agglomeration was very similar on weekdays and weekends. However, the coverage of high visitor density on weekdays was greater than it was on weekends. 

**Figure 4:**
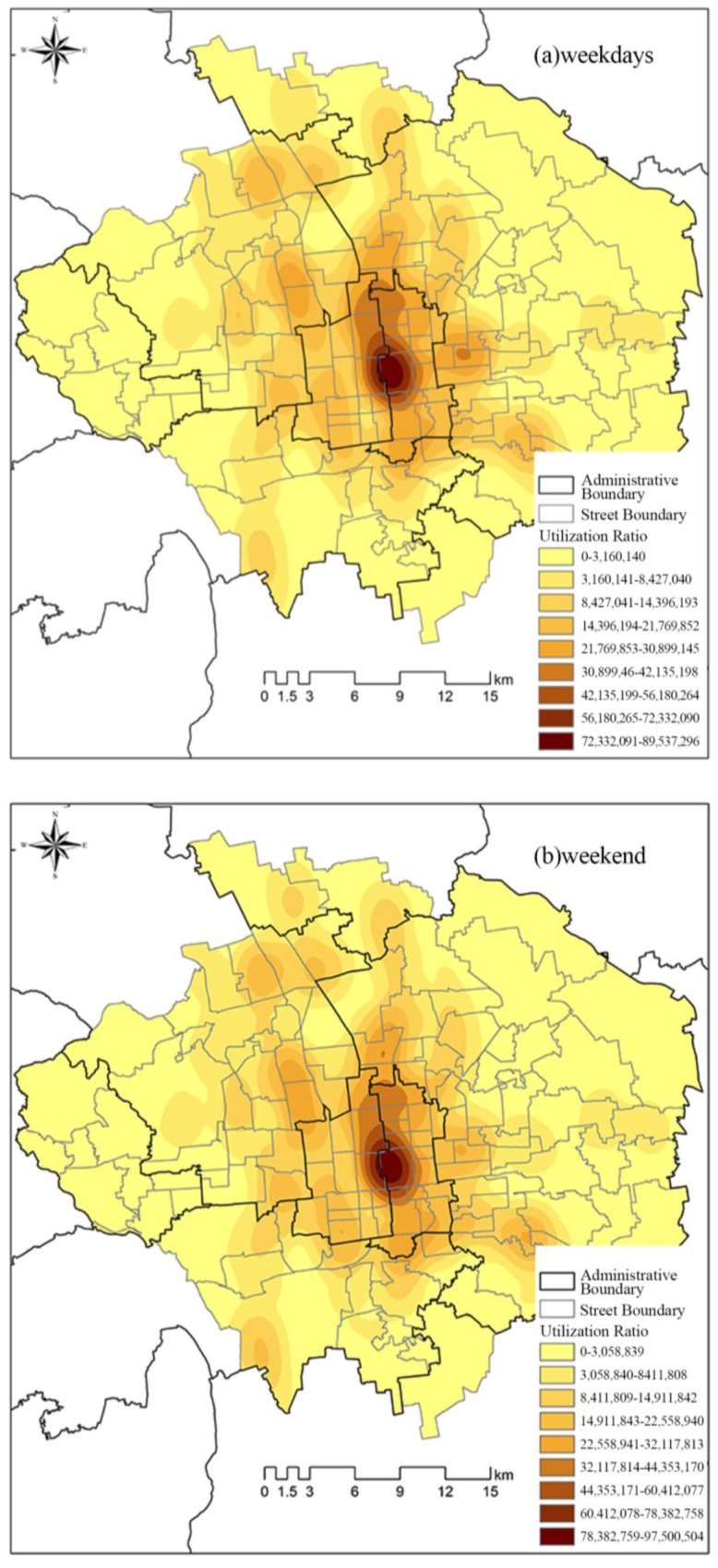
Utilization ratio of urban green spaces on (**a**) weekdays and (**b**) weekends.

As discussed above, the spatial pattern of the distribution of visitors was reflected in the number of visitors. The KDE was applied to obtain the utilization ratio of urban green spaces for visitors on weekdays or weekends. Consequently, the spatial structure of the distribution of visitors per unit area could be determined. Figure 4 shows that only one single-core agglomeration comprising Beihai Park, Jingshan Park, and Zhongshan Park was evident in central Beijing on weekdays and weekends. Moreover, the kernel density value relating to visitor numbers per unit area decreased progressively from the core area to the suburbs. The relatively high value of the kernel density relating to visitor numbers per unit area distributed along Third Ring Road was notable.

**Figure 5:**
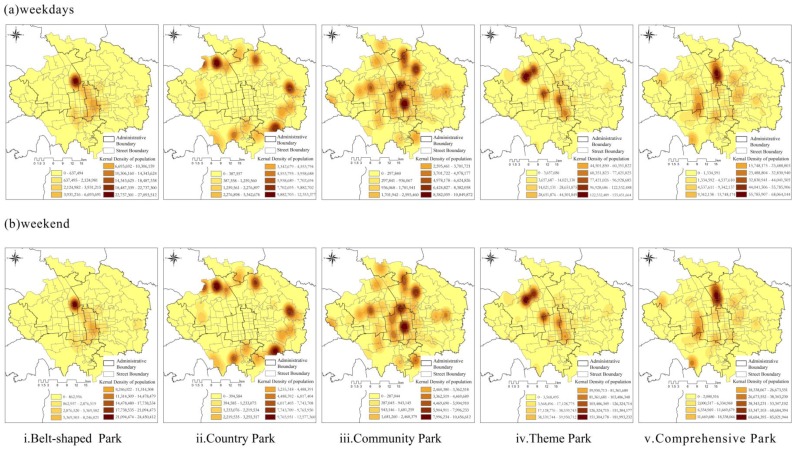
Kernel density of visitor numbers in the five kinds of urban green spaces during (**a**) weekdays and (**b**) weekends.

The kernel density core of the distribution of visitors among the five types of urban green spaces appeared in the same site on weekdays and weekends. However, there were significant differences in the values of the kernel density for these five types. Figure 5a reveals that only one core of visitors’ distribution in theme parks was apparent in Yuan Dynasty City Wall Relics Park. Furthermore, the kernel density of visitors in theme parks was relatively higher on weekdays than on weekends. The distribution of visitors in country parks constituted several cores around Five Ring Road, with the four largest cores being distributed in Shucun Country Park, Qing heying Country Park, Dongba Country Park, and Laojuntang Country Park (Figure 5b). The kernel density of visitors in country parks was evidently higher during weekends compared with weekdays, indicating that country parks provided an important recreation space during weekends. The kernel density core of visitors’ distribution in community parks was the primary component of the five dual-core structures that were relatively well-distributed within the central part of the city. Relatively high kernel density cores were evident in Changpu River Park, Rendinghu Park, Liuyin Park, Wanghu Park, North Creek Park, and Lishuiqiao Park (Figure 5iii). Moreover, the kernel density values of visitors in community parks were higher during weekdays than on weekends. The kernel density core of visitor distribution in theme parks was mainly located in Dongcheng, Xicheng, and Haidian Districts, entailing single-core as well as dual-core structures (Figure 5iv). The kernel density core of visitors’ distribution in comprehensive parks was mainly located along Five Ring Road and approximated a single-core structure (Figure 5v). It should be noted that the kernel density of visitors in comprehensive parks was higher on weekends than on weekdays.

### 3.3. External Factors Influencing Visitors’ Distribution in Urban Green Spaces

A question that emerged was whether the spatial patterns of visitors in urban green spaces were associated with the external factors, and to what extent. The $P_{D,H}$ values reflecting the external factor’s effect on the density of visitors in the five types of urban green spaces were measured using geographical detectors methods (Table 4). 

The $P_{D,H}$ values of geographical detectors indicated that the spatial distribution of visitors in the five types of urban green spaces was differentially associated with transportation convenience as follows: country parks (0.32) > comprehensive parks (0.25) > belt-shaped parks (0.22) > theme parks (0.16) > community parks (0.10). Transportation convenience had the greatest correlation value with the use of country parks, because these parks, which are mostly distributed around Five Ring Road at a distance from the city center, are mainly accessed by visitors using public transportation. Transportation convenience also had a significant correlation value with the use of comprehensive parks. This may be because comprehensive parks, which are well-distributed in central Beijing, are accessed by residents of the entire city through public transportation. Transportation convenience had the lowest correlation value with the use of community parks which were mainly used by local residents by walking. Additionally, the use of belt-shaped parks and theme parks had the least remarkable correlation with transportation convenience.

The $P_{D,H}$ values of geographical detectors indicated the following effects of population density on the spatial distribution of visitors in the five types of urban green spaces: country parks (0.52) > belt-shaped parks (0.26) > community parks (0.22) > theme parks (0.19) > comprehensive parks (0.17). Thus, the correlation value of population density was the highest with country parks and the lowest with comprehensive parks. Country parks are mainly located along the edge of Five Ring Road and are used for recreation by nearby residents. With fewer residents from central Beijing, their uses have a great correlation with population density. However, comprehensive parks with large service radiuses are well-distributed in central Beijing and can, therefore, be used by residents of the entire city. Consequently, population density had the least remarkable correlation with the use of comprehensive parks. 

The $P_{D,H}$ values of geographical detectors indicated the following ranking of the effects of commercial facilities on the spatial distribution of visitors in the five types of urban green spaces: country park (0.68) > belt-shaped parks (0.29) > theme parks (0.25) > comprehensive parks (0.21) > community parks (0.18). The spatial distribution of visitors in country parks had the most remarkable correlation with commercial facilities caused by the limited presence of commercial facilities around country parks. The effects of commercial facilities on other types of urban green spaces were almost the same because of the presence of several commercial facilities in their vicinity in central Beijing. It should be noted that the $P_{D,H}$ values of commercial facilities were higher than those for transportation convenience and population density in relation to country parks, belt-shaped parks, and theme parks. This suggested that commercial facilities were the dominant factor influencing the spatial distribution of visitors in urban green spaces.

Thus, the ranking of the correlation of each factor with the spatial distribution of visitors to urban green spaces was as follows: commercial facilities (0.24) > population density (0.18) > transportation convenience (0.16). Commercial facilities were evidently the most influential factor, while transport facilitation had the least remarkable correlation with the usage of urban green spaces. The transportation factor only had a small impact in areas with the worst transportation conditions, indicating that visitors’ choices of green spaces do not rely on the mode of transportation in mega-cities such as Beijing. The presence of commercial facilities around the park was a decisive factor associated with the spatial distribution of visitors. This finding is in line with the increasing and simultaneous demand for both service and recreation facilities. 

## 4. Discussion

In Beijing, some green spaces are always crowded, while others have only few visitors. By confirming whether external factors are influential on the use of green space, this study aims to figure out strategies to balance the use of green spaces, especially to improve the utilization of the less used ones. In this study, we analyzed the spatiotemporal pattern of visitors’ distribution in urban green spaces in central Beijing. Focusing on external factors that are associated with on visitor distribution in these spaces, we examined the correlation of transportation convenience, population density, and commercial facilities with the spatial distribution of visitors in urban green spaces. Our conclusions can be summarized as follows.

Firstly, there are significant differences in the spatial distribution characteristics of urban green spaces in central Beijing. We found that the degree of spatial agglomeration was highest for community parks. Conversely, it was lowest for belt-shaped parks. The degree of spatial agglomeration of theme parks was relatively high, while comprehensive parks were found to be well-distributed within central Beijing. The distribution of country parks demonstrated the gradual formation of a “park ring” around Five Ring Road. 

Secondly, the spatiotemporal pattern of visitors’ distribution within urban green spaces also showed considerable variations. From the temporal perspective, the number of visitors in urban green spaces showed evident variation during both weekdays and on weekends. From the spatial perspective, the degree of spatial agglomeration in urban green spaces was higher on weekdays than on weekends. Specifically, spatial agglomeration was highest in comprehensive parks on weekdays, while it was highest in theme parks on weekends. Moreover, application of the KDE method revealed that the spatial structure of the distribution of visitors evidenced two dual-core agglomerations combined with two single-core agglomerations. 

Thirdly, the number of visitors visiting urban green spaces in central Beijing was found to be closely correlated with transportation convenience, population density, and commercial facilities. From our findings, we can conclude that commercial facilities have the greatest influence on the distribution of visitors in urban green spaces, followed by population density. Transportation convenience was found to have the least influence on the distribution of visitors in urban green spaces.

To the best of our knowledge, this study is the first study that applied app data on a large scale to examine the use of urban green spaces in relation to external factors in Beijing. The wide spatial coverage of the study yielded valuable information that can facilitate the planning and design of urban green spaces in other megacities. The following points for consideration emerged from the study. Firstly, given the incidence of urban sprawl in megacities such as Mexico City, new urban green spaces should be established to provide a natural environment. Better land use policies can be formulated through a consideration of external factors that influence the use of urban green spaces when selecting sites for their development [44]. Because commercial facilities have the greatest correlation with the distribution of visitors in urban green spaces, it is important when planning new urban green spaces to choose locations in proximity to recreational business districts, especially in those areas where there is a need to enhance the use of green spaces. A second point relates to the fact that there are few possibilities for adding new green spaces in crowded megacities across the world such as Tokyo and the old city of Beijing. Consequently, the emphasis should be on improving land use and creating open spaces with a high utilization ratio. Attached green space within highly accessible residential areas that also have commercial facilities should be a priority within future urban greening programs. Thirdly, urban green spaces have considerable potential for promoting an active and healthy lifestyle [23]. There is significant potential to promote moderate to vigorous physical activities among urban residents through the effective use of urban green spaces. Our findings provide important insights for planning active city landscapes. Previous studies have shown that internal factors such as living contexts, vegetative quality, and the accessibility of green spaces affect the use of urban green spaces for physical activities [23]. However, this study has demonstrated the significant correlation that exists between external factors such as transportation convenience, population density, commercial facilities and density of visitors in urban green spaces. These findings highlight the importance of focusing on the use of urban green spaces to improve the health of urban residents worldwide.

## 5. Conclusions

Urban green spaces are places where individuals can engage in physical activities to improve their health. Studies that explore the relationship between urban green spaces and their use are, therefore, of significance for improving human health. For this study, we used app data to explore the spatiotemporal pattern of use of urban green spaces and the external factors contributing to their use in central Beijing. The findings of the study revealed that traffic convenience, population density, and commercial facilities are all associated with the use of green spaces. These findings will facilitate the planning of urban green spaces as well as enhance the use of previously underutilized urban green spaces. The recommendations derived from our study are as follows.

The layouts of urban green spaces should be adjusted and their structures should be optimized. The number of belt-shaped parks was far fewer than other kinds of green spaces. Considering the paucity of belt-shaped parks that can be used by visitors as corridors connected with other urban green spaces, a reasonable option would be to increase the number of belt-shaped parks located between other urban green spaces. Numbers of community and theme parks are relatively high, and their utilization ratios are also very high in areas where they easily form aggregations. Therefore, we recommend adding transportation facilities to improve the use of theme parks where their utilization is low. Comprehensive parks and country parks are mainly accessed by visitors using public transportation. Consequently, enhancing their accessibility is critical to increase their utilization ratio. Moreover, the western suburbs have abundant green spaces, while Second Ring Road and the southern part of central Beijing are poorly endowed with these resources. The southern part of central Beijing is a component of the old city which does not have new areas of construction land. We, therefore, recommend taking full advantage of the attached green space to cover the shortage of urban green spaces in this part of the city. In order to make urban green spaces more effective, similar studies in more megacities can be conducted to help us recognize common issues that affect the use of urban green spaces.

## Figures and Tables

**Table 1:** Statistics related to urban green spaces in central Beijing.

Belt-Shaped Park		Country Park		Community Park		Theme Park		Comprehensive Park	
Number	Area (km^2^)	Number	Area (km^2^)	Number	Area (km^2^)	Number	Area (km^2^)	Number	Area (km^2^)
17	1.7	40	15.76	58	4.95	61	22.72	32	20.72

**Table 2:** Gini coefficient of urban green spaces in Beijing.

	Belt-Shaped Park	Country Park	Community Park	Theme Park	Comprehensive Park
Gini Coefficient	0.300	0.405	0.466	0.414	0.399

**Table 3:** Gini coefficient of visitor numbers in urban green spaces in central Beijing.

Types of Urban Green Spaces	The Gini Coefficient	
	Weekday	Weekend
The belt-shaped park	0.330	0.237
The country park	0.319	0.260
The community park	0.297	0.240
The theme park	0.338	0.302
The comprehensive park	0.340	0.286

**Table 4:** $P_{D,H}$ values of the external factors.

Types of Urban Green Spaces	Traffic Convenience	Population Density	Commercial Facilities
The belt-shaped park	0.22	0.26	0.29
The country park	0.32	0.52	0.68
The community park	0.10	0.21	0.18
The theme park	0.16	0.19	0.25
The comprehensive park	0.25	0.17	0.21
The total	0.16	0.18	0.24
